# The study of women, infant feeding and type 2 diabetes after GDM pregnancy and growth of their offspring (SWIFT Offspring study): prospective design, methodology and baseline characteristics

**DOI:** 10.1186/s12884-015-0587-z

**Published:** 2015-07-17

**Authors:** Erica P. Gunderson, Shanta R. Hurston, Kathryn G. Dewey, Myles S. Faith, Nancy Charvat-Aguilar, Vicky C. Khoury, Van T. Nguyen, Charles P. Quesenberry

**Affiliations:** Division of Research, Kaiser Permanente Northern California, 2000 Broadway, 94612-2304 Oakland, CA USA; Department of Nutrition, University of California, Davis, One Shields Ave, 95616 Davis, CA USA; Department of Nutrition, Gillings School of Global Public Health, University of North Carolina, Chapel Hill, USA

**Keywords:** Gestational diabetes mellitus, Breastfeeding, Infant growth, Obesity, Temperament, Type 2 diabetes mellitus, Prospective study

## Abstract

**Background:**

Breastfeeding is associated with reduced risk of becoming overweight or obese later in life. Breastfed babies grow more slowly during infancy than formula-fed babies. Among offspring exposed *in utero* to maternal glucose intolerance, prospective data on growth during infancy have been unavailable. Thus, scientific evidence is insufficient to conclude that breastfeeding reduces the risk of obesity among the offspring of diabetic mothers (ODM).

To address this gap, we devised the Study of Women, Infant Feeding and Type 2 Diabetes after GDM Pregnancy and Growth of their Offspring, also known as the SWIFT Offspring Study. This prospective, longitudinal study recruited mother-infant pairs from the SWIFT Study, a prospective study of women with recent gestational diabetes mellitus (GDM). The goal of the SWIFT Offspring Study is to determine whether breastfeeding intensity and duration, compared with formula feeding, are related to slower growth of GDM offspring during the first year life. This article details the study design, participant eligibility, data collection, and methodologies. We also describe the baseline characteristics of the GDM mother-infant pairs.

**Methods:**

The study enrolled 466 mother-infant pairs among GDM deliveries in northern California from 2009–2011. Participants attended three in-person study exams at 6–9 weeks, 6 months and 12 months after delivery for infant anthropometry (head circumference, body weight, length, abdominal circumference and skinfold thicknesses), as well as maternal anthropometry (body weight, waist circumference and percent body fat). Mothers also completed questionnaires on health and lifestyle behaviors, including infant diet, sleep and temperament. Breastfeeding intensity and duration were assessed via several sources (diaries, telephone interviews, monthly mailings and in-person exams) from birth through the first year of life. Pregnancy course, clinical perinatal and newborn outcomes were obtained from health plan electronic medical records. Infant saliva samples were collected and stored for genetics studies.

**Discussion:**

This large, racially and ethnically diverse cohort of GDM offspring will enable evaluation of the relationship of infant feeding to growth during infancy independent of perinatal characteristics, sociodemographics and other risk factors. The longitudinal design provides the first quantitative measures of breastfeeding intensity and duration among GDM offspring during early life.

## Background

Offspring exposed to maternal diabetes *in utero* have an increased risk of becoming overweight or obese, and developing impaired glucose tolerance, the metabolic syndrome, or type 2 diabetes [1–8]. Both exposure to maternal metabolism during fetal life and postnatal feeding practices may exert influences on the programming of the offspring’s growth and future disease risk [9–11]. Breastfeeding may have beneficial effects on the long-term health of the offspring of diabetic mothers (ODM), but evidence is limited to primarily retrospective studies that have not characterized the intrauterine metabolic environment, and have not measured infant growth or controlled for other key risk factors that predict obesity-related metabolic diseases.

Postnatal feeding greatly influences early growth and metabolism in animals and humans. In animals, overfeeding during early life is associated with higher adiposity in adolescence [12], and metabolic adaptations [13, 14]. In humans, formula milk feeding compared with breastfeeding has shown “growth-accelerating effects” on both length and weight gain throughout infancy that manifest as a strong dose–response gradient from 3–6 months of age [15]. Breastfed infants grow more slowly than formula-fed infants [15, 16], show a greater decline in weight-for-length (WLZ) z-score between 3 and 12 months [17, 18], and tend to be leaner by 12–18 months [19]. From 5 months of age, the percentage of body fat tends to decline among breastfed infants, but actually increases among formula fed infants [17, 18]. However, others who found lower percent body fat mass for exclusively breastfed versus formula fed infants during the first year of life reported that these differences in body composition did not persist to 24 months of age [20].

One explanation for these differences in body fat could be the lower energy intake among breastfed infants and the heightened ability to regulate their intake in response to internal satiety cues [21]. Another possibility is that breast milk composition includes bioactive substances that may regulate energy balance and fat deposition, including the lower protein content relative to formula milk. In fact, formula-fed neonates have higher blood insulin levels than breastfed newborns [22–24], and this higher protein intake can produce higher insulin secretion [23]. Dietary protein intake during infancy has been directly associated with higher BMI at older ages [25–27].

In the general population, breastfeeding is associated with a lower risk of becoming overweight or obese during childhood and adolescence [28], even after accounting for maternal obesity and family lifestyle behaviors [29–32]. In 2007, a consensus report based on 40 years of research, mostly in Caucasians, concluded that breastfeeding is linked to a 22–24 % lower risk of child and adolescent overweight and obesity [30–34], and that associations are stronger with exclusive breastfeeding [32], independent of dietary and physical activity patterns later in life [31]. The evidence is based largely on observational studies with the potential for unmeasured confounding. Yet, randomization of individual infants to exclusive breastfeeding or formula feeding is not desirable or feasible. Therefore, a causal link to child obesity has not been established [35, 36].

Results of a large intervention trial to promote breastfeeding have challenged the evidence that breastfeeding prevents child obesity [37]. Kramer and colleagues conducted a cluster-randomized trial in Belarus to increase exclusive breastfeeding rates and evaluate the impact of breastfeeding on child obesity [38]. The study found no differences in infant growth or child adiposity at age 6.5 years associated with the intervention to promote breastfeeding [39, 40]. However, the study population was characterized by a much lower prevalence of child overweight and obesity than found in the U.S. The study also could not make comparisons between breastfeeding versus formula feeding, as most studies of infant growth have reported. Furthermore, the study did not consider the impact of maternal gestational glucose intolerance on infant growth parameters because screening for GDM was not performed in Belarus during the study period. Thus, these study findings may not be generalizable to other settings or high-risk populations, and demonstrate the need for studies that employ quantitative measures of breastfeeding intensity, and carefully control for perinatal and postnatal exposures and other determinants of body size particularly among high-risk groups.

Other researchers have proposed that “genetic and environmental determinants such as socioeconomic status, parental obesity, smoking, birth weight, and rapid infancy weight gain far supersede infant-feeding practices as risk factors for childhood obesity” [41]. It is important to note that studies to examine rapid weight gain in early life [42, 43] have rarely controlled for infant feeding practices, or the intrauterine maternal metabolic milieu [44–46]. Thus, prospective studies are needed that evaluate both prenatal and postnatal exposures, specifically breastfeeding intensity, determinants of rapid weight gain, maternal metabolic status, infant behaviors and intake of supplemental foods [47]. Emerging evidence about breast milk composition and the variability in infant growth may support a causal link with subsequent child obesity [26]. However, analyses of sibling pairs who share genetics and environmental exposures have attributed the association between breastfeeding and child overweight to unmeasured confounding by the mothers’ choice of feeding method or other risk factors with greater effects on body size [48].

First, we critically review the evidence that maternal glucose tolerance during gestation and postnatal feeding are associated with future adiposity and health outcomes of ODM. Secondly, we evaluate the scientific evidence that breastfeeding influences the future adiposity and diabetes risk among ODM, including GDM offspring. Finally, we detail the study design, and methodologies for the Study of Women, Infant Feeding and Type 2 Diabetes after GDM pregnancy (SWIFT) and Growth of Their Offspring, also known as the SWIFT Offspring Study. This longitudinal study recruited 466 mother-infant pairs exposed to GDM pregnancy and prospectively assessed their feeding practices monthly and growth from birth to one year of age.

### Maternal glucose intolerance and intrauterine effects on offspring growth patterns

Intrauterine metabolism may strongly influence perinatal and postnatal growth [4, 49, 50]. Maternal diabetes and obesity are independently associated with newborn macrosomia [51, 52]. Among macrosomic GDM offspring, higher adiposity persists through the first year of life [50]. GDM offspring grow more slowly after birth through the first 1–2 years of life with rapid weight gain thereafter [6, 49], and are more likely to become overweight. [4, 5, 53] This slowed postnatal growth pattern is called “catch-down” growth, and refers to a growth pattern during infancy subsequent to exposure of the fetus to excess nutritional or metabolic substrates in utero [54]. This early growth pattern is strongly influenced by a drive to compensate for the intrauterine effects on the fetus of the maternal metabolism associated with obesity and glucose intolerance [55]. It is not known whether postnatal feeding method, (i.e., breastfeeding compared with formula feeding) further slows postnatal growth among GDM offspring. Silverman et al. examined infant growth patterns from birth to 6 months in GDM offspring [6], but did not evaluate breastfeeding. However, Thomas et al. found smaller increases in fat mass and percent body fat from birth to 4 months in GDM versus non-GDM offspring adjusted for breastfeeding [56].

Among GDM offspring, the relative importance of intrauterine and postnatal risk factors in relation to infant growth and development of future overweight and chronic disease is not well understood. Prospective studies are needed to evaluate the association of breastfeeding with infant growth in GDM offspring controlling for the intrauterine environment and other correlates of obesity.

### Breastfeeding and risk of diabetes for offspring of diabetic mothers (ODM)

Evidence is less available about the role of breastfeeding on future disease risk among the offspring of diabetic mothers (ODM), including those born to women with GDM or pre-gestational diabetes (Type 1 or Type 2 diabetes mellitus). Both animal and human studies show that postnatal feeding plays a critical role in the programming of body adiposity, obesity, and glucose tolerance among ODM [14, 57–59]. Among high-risk indigenous populations, breastfeeding has been associated with a 50–75 % lower risk of type 2 diabetes. Pettit et al. reported a lower incidence of type 2 diabetes among Pima Indians (age 10–39 years) who were breastfed exclusively for 6–9 weeks adjusted for age, sex, parental diabetes, and birth weight. [60] When stratified by exposure to maternal diabetes *in utero*, those not exposed had a 50 % lower prevalence of type 2 diabetes associated with exclusive breastfeeding for >2 months versus none, but the small sample size for the ODM limited the statistical power [61].

Two case–control studies of ODM both reported lower risk of type 2 diabetes with breastfeeding or longer breastfeeding duration. The multi-ethnic U.S. study SEARCH of 80 youth (aged 10–21 years) with incident type 2 diabetes found a 57 % lower odds of ever being breastfed for cases versus controls after adjustment for 12 potential confounders [62]. Among indigenous Canadian youth <18 years, breastfeeding ≥12 months was associated with lower odds of type 2 diabetes (OR = 0.24; 95 % CI:0.13-0.84) adjusted for maternal type 1 diabetes or GDM and covariates [63].

### Breastfeeding, growth and weight status in the offspring of diabetic mothers (ODM)

Breastfeeding may lower risk of childhood overweight and obesity in ODM. A non-randomized longitudinal study of German ODM (83 type-1 diabetes, 29 GDM) reported that intake of breast milk from a diabetic mother versus banked donor breast milk [64, 65] during the first week of life was associated with a 2-fold higher (OR = 1.91; 95 % CI: 1.10-3.30) risk of becoming overweight at age 2 years [64]. However, breast milk intake after the first week of life and duration of breastfeeding were not associated with the risk of child overweight adjusted for early neonatal breast milk intake [65]. Given that subjects were not randomized, potential confounding and reverse causation (i.e., unknown indication for feeding banked breast milk) cannot be excluded. Furthermore, total volume of milk consumed, and maternal glycemic control were not evaluated in the ODM [66].

In several retrospective studies of ODM, inverse associations between breastfeeding and body adiposity or risk of overweight in childhood have been reported. Among pre-adolescent ODM aged 6–13 years (*n* = 85), >6 vs. ≤6 “breast milk” months (weighted months of mixed feeding) was associated with lower overall body adiposity [67]. A second study of ODM (*n* = 94) reported slower growth at ages 4–6 years and 6–9 years in the breastfed group (sufficient number of “breast-milk months”) compared with low breast-milk group [68]. The Nurses’ Health Study of Offspring examined youth aged 9–14 years who were ODM (419 GDM, 56 pre-gestational diabetes) and found a lower risk of becoming overweight associated with “ever” breastfeeding [69]. A German study of 324 GDM offspring aged 2–8 years found that exclusive breastfeeding for 3 months or longer was associated with a lowered risk of becoming overweight (0.55, 95 % CI:0.33-0.91), but only for offspring of obese GDM mothers [70]. These data are suggestive of protective effects of breastfeeding, but have not controlled for multiple postnatal confounders.

These studies rely on later recall of duration or exclusivity, lack quantitative data on breastfeeding intensity, and did not measure infant growth or postnatal attributes. Although a few studies have controlled for characteristics of the intrauterine milieu (i.e., GDM severity, gestational weight gain), almost none have prospectively assessed infant growth, breastfeeding intensity, and other behaviors from birth through childhood. Finally, previous studies have never prospectively examined infant behaviors and growth from birth throughout infancy and toddler ages among GDM offspring.

### Research gaps

In developed countries, scientific evidence indicates a robust, albeit modest, association between breastfeeding and the lower risk of being overweight during childhood and adolescence in the general population, even after accounting for maternal obesity and family lifestyle behaviors [71]. However, because the studies are observational, the findings may be due to bias from unmeasured confounding, as suggested by the null findings from one study that utilized a sibling design and another that randomized clinics to a breastfeeding promotion intervention [47]. Even less is known about whether breastfeeding confers similar protection against obesity and type 2 diabetes for ODM given the retrospective designs where reverse causation cannot be ruled out. In ODM, findings are mixed (i.e., higher, lower, or no difference) as to whether breastfeeding influences the risk of overweight in childhood or adolescence. However, some studies also report that breastfeeding may be associated with relatively less body fat in older ODM children based on history of a sufficient number of “breast milk months”.

Overall, the limited epidemiologic evidence supports the benefits of breastfeeding for ODM. Most studies are retrospective and rely on recall of breastfeeding in older children or adults, and have not prospectively evaluated breastfeeding intensity, or measured growth during infancy. Retrospective studies measured weight and height among school age children or adolescents, and asked mothers to recall breastfeeding, including exclusive breastfeeding, up to more than a decade later. These studies did not fully control for the fetal metabolic milieu (e.g., the severity of GDM, or type of treatment) or infant feeding practices. Prospective studies have limited sample sizes (<100 ODM cases) and are characterized by wide heterogeneity in the type of maternal diabetes, as well as limited data on pregnancy course and perinatal outcomes (i.e., infant size and health status at birth) and lack measurements of infant growth. Thus, for GDM offspring, studies are needed that evaluate quantitative breastfeeding measures and infant growth independent of potential confounders, such as parental obesity, socio-demographics, intrauterine metabolic milieu and postpartum glucose tolerance as well as other infant feeding behaviors [72–74]. This is especially important given that infants and toddlers in the general population are often consuming excess fruit juice and sugary beverages, candies, and insufficient fruit and vegetables [75].

The American Academy of Pediatrics recommends that all infants should be exclusively breastfed through 6 months of age and that breastfeeding should continue until the infant is 1 year of age. Although 80 % of US women initiate lactation, only 45 % percent report “any” breastfeeding at 6 months and less than 20 % report “exclusively” breastfeeding their infants at 6 months [76]. Thus, increasing breastfeeding has substantial potential for positive effects on infant and maternal health in the general population. The evidence is insufficient to develop evidence-based public health recommendations regarding the impact of breastfeeding on future health outcomes for high-risk infants and their mothers.

To address these gaps in knowledge, we implemented the SWIFT Offspring study, a prospective study of growth during infancy among GDM offspring that evaluates the associations with breastfeeding intensity based on quantitative methods independent of the intrauterine environment, perinatal outcomes and postnatal behaviors related to serial growth measures from birth through one year of age.

### The SWIFT offspring study design and aims: prospective cohort of GDM offspring

The overall goal of the SWIFT Offspring study is to determine whether intensive breast feeding compared to intensive formula feeding is related to slower infant weight gain in the first year of life, and to lower weight retention in mothers at one year postpartum. The study enrolled 466 GDM mother-infant pairs and followed them prospectively from birth to 12 months of age. Data collection occurred at three in-person exams (6–9 weeks, 6 months and 12 months of age), and via infant feeding diaries that mothers completed from birth to 4–6 weeks, telephone interviews at 4–6 weeks postpartum, and monthly mailed surveys from 3–11 months from the parent SWIFT Study [77]. Self- and interviewer administered questionnaires were used to evaluate breastfeeding intensity and duration and other covariates, including the intrauterine milieu (maternal BMI, gestational weight gain, prenatal glucose tolerance, type of GDM treatment), paternal BMI, family history of diabetes, socio-demographics, newborn outcomes (gestational age, sex, NICU admission, length of stay, birth weight and size for gestational age at birth), sleep habits, dietary intake and temperament. Anthropometric measurements were obtained in mother-infant pairs at each exam utilizing standardized research protocols.

This SWIFT Offspring Study evaluates the independent associations of infant feeding characteristics and growth during the first year of life within a large racially and ethnically diverse, contemporary cohort of GDM infants (72 % minority, 25 % low income; ≤185 % of the federal poverty level enrolled in the WIC program). The study protocol, materials and procedures were approved by the Institutional Review Board at Kaiser Permanente Northern California. The SWIFT Offspring Study (2009–2013) was funded by the American Diabetes Association.

The study specific aims are to determine whether intensive breastfeeding, compared to intensive formula feeding, is associated with:

- Aim 1. Slower rate of weight gain (change in weight for age z-score) from birth to 6–9 weeks and from 2 to 6 months of age in the offspring;
- Aim 2. Lower weight for length z-score, and smaller waist girth and skinfold thicknesses at 12 months of age in the offspring; and
- Aim 3. Lower BMI and smaller waist girth at 6 months and 12 months postpartum in mothers.

## Methods

### Study participants and setting

The SWIFT Offspring Study cohort is an observational study of 466 mother-infant pairs recruited from among women with recent GDM who were participants in the SWIFT Study [77]. The parent SWIFT Study enrolled 1,035 women diagnosed with GDM by the Carpenter and Coustan criteria during pregnancy and who delivered a singleton, pregnancy of ≥35 weeks gestation at a Kaiser Permanente Northern California (KPNC) hospital from August 2008 through December 2011. The study design and recruitment protocol for the SWIFT Study have been described in detail elsewhere [77].

**Fig. 1:**
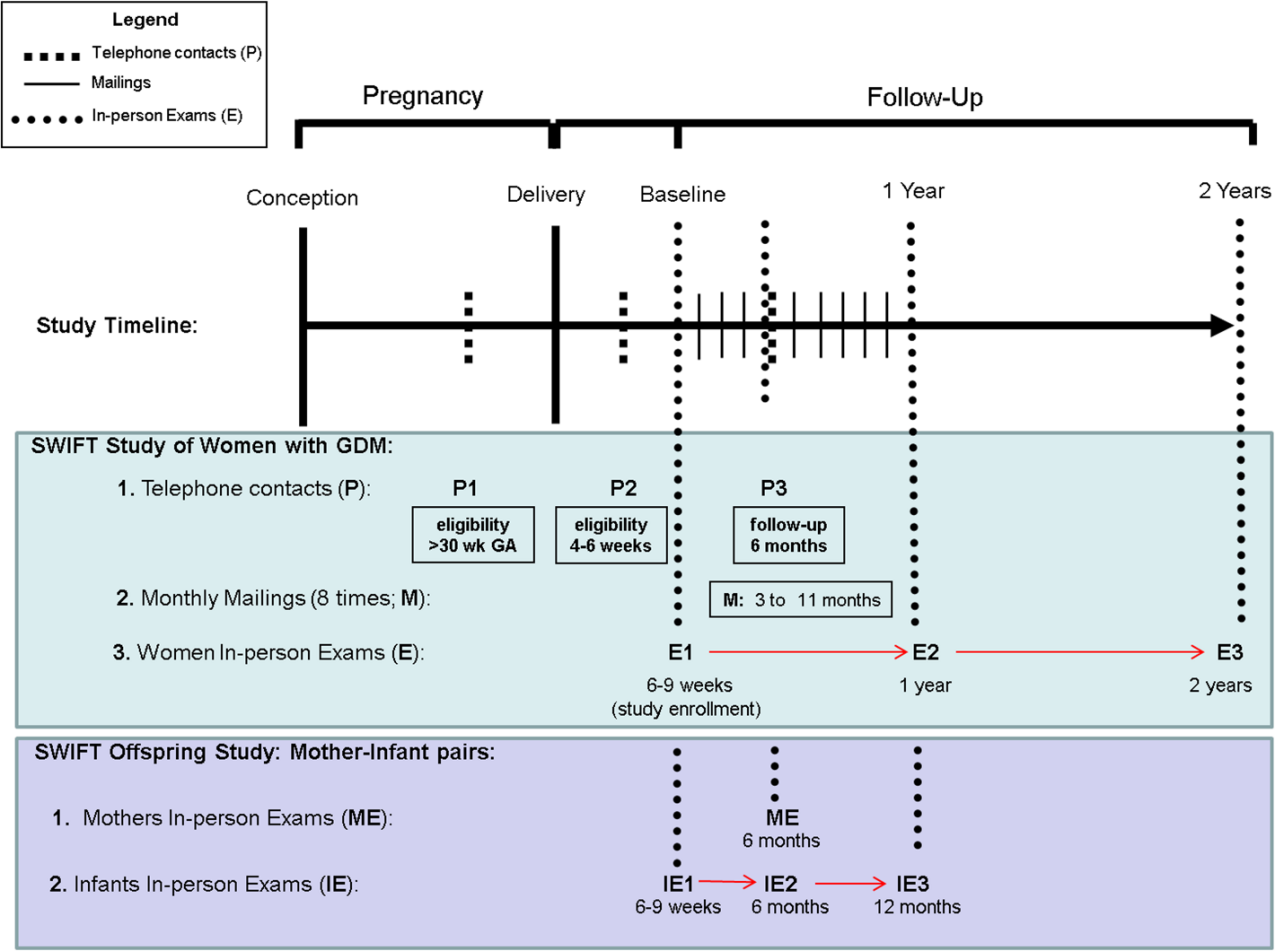
Diagram of the Parent SWIFT Study and SWIFT Offspring Studies Coordinated Data Collectin Timelines for Women and their Offspring

A description of the coordinated in-person exams (E) for the parent SWIFT Study and the in-person infant exams (IE) and additional maternal exams (ME) for the SWIFT Offspring Study are shown in Fig. 1.

From August 2009 to December 2011, the SWIFT Offspring Study enrolled 466 mother-infant pairs after mothers provided written informed consent for their own and the infants’ participation in three in-person exams conducted at 6–9 weeks (baseline), 6 months and 12 months of age (Fig. 1). The study employed standardized protocols and research quality calibrated instruments to measure infant head circumference, weight, supine length, abdominal circumference and three skinfold thicknesses. Mothers completed questionnaires that gathered data on breastfeeding and formula feeding, and infant health, development, supplemental dietary intake, sleep habits, temperament and other behaviors, as well as reported paternal weight and height, reproductive and health history, inter-current pregnancies, perinatal outcomes, postpartum depression and lifestyle behaviors (i.e., smoking, sleep habits, physical activity and dietary intake) during the postpartum period. Saliva specimens were also collected from infants.

### Recruitment and eligibility criteria – the parent SWIFT study

SWIFT participants were recruited from 12 Kaiser Permanente Northern California (KPNC) medical centers and medical office facilities in 2008–2011 throughout the 5,000 square mile KPNC region. The SWIFT Offspring study recruitment and follow-up began in August 2009 and follow up continued through December 2013. Eligibility criteria were as follows: a healthy, live born singleton infant ≥35 week’s gestation and birth weight of ≥2,500 g born to mothers enrolled in the SWIFT study. Infants with one or more serious medical condition(s) (e.g., failure to thrive, physical impairment affecting feeding ability, chronic infectious disease, severe jaundice, or other metabolic disorder) were excluded. Mothers provided written informed consent for their infants after being fully informed of all aspects of the study, including potential risks and benefits, and were given an additional incentive for their child’s participation at each study exam. Characteristics of the SWIFT Offspring study of mother-infant pairs (*n* = 466) are displayed in Table 1.

**Table 1:** Maternal and offspring (*n* = 466 mother-infant Pairs) characteristics (enrolled 2009–2011)

Newborn	N, (%)	Mother	N, (%)
Sex		Education	
Female	210 (45.1)	High School or less	118 (25.4)
Male	256 (54.9)	Some college	134 (28.8)
Gestational age (weeks)		4 years of college or more	213 (45.8)
35-36 preterm	15 (3.2)	Pre-pregnancy BMI (kg/m^2^)	
37-40 term	381 (81.8)	Obese ≥ 30	206 (44.3)
>40	70 (15.0)	Overweight = 25–29.9	137 (29.5)
Size for Gestational Age		Prenatal WIC enrollment (Y)	130 (27.9)
SGA	9 (1.9)	Infant Feeding (since birth)	
AGA	332 (71.3)	Exclusive Breast milk	123 (26.4)
LGA	125 (26.8)	Mostly Breast milk	188 (40.3)
Apgar Score		Mostly Formula or inconsistent	84 (18.0)
1 min(≥7)	433 (93.3)	Exclusive Formula	71 (15.2)
5 min(≥7)	58 (98.9)	Race/ethnicity	
Birth weight categories		White	131 (28.1)
<2500 g	11 (2.4)	Black	38 (8.2)
2500 to 3499 g	244 (52.4)	Hispanic	165 (35.4)
3500 to 4000 g	150 (32.2)	Asian	121 (26.0)
>4000 g	61 (13.1)	Other	11 (2.4)
	Mean (SD)		Mean (SD)
Gestational age (weeks)	39.0 (1.1)	Gestational weight gain (kg)	10.1 (7.0)
Birth weight (g)	3438.7 (502.8)	Age (years)	33.1 (4.7)
Length (cm)	50.7 (2.3)	Parity	2.2 (1.2)
Infant WLZ-score (ages)		Maternal 6–9 weeks Postpartum	
Birth	−0.33 (1.35)	Waist circumference (cm)	91.7 (14.6)
6-9 weeks	0.18 (1.19)	Weight (kg)	79.3 (20.3)
6 months	0.29 (1.06)	BMI (kg/m^2^)	30.8 (6.9)
12 months	0.41 (1.07)	HOMA-IR	5.9 (4.3)

### Sample size and power analysis

Minimum detectable differences in means (either at exam or change between exams) of a continuous variable (e.g., change in weight-for-age z-score, skinfold thickness, waist girth) are expressed in standard deviation units, in analyses of offspring and mothers at 6–9 weeks (offspring only, Aim 1), at 6 months and at 12 months (Table 2). These estimates are based on the standard t-test for differences in means, in a comparison of breastfeeding (66.7 %) vs. none/minimal (33.3 %). In Aim 1, for analyses of infants, we have sufficient power (0.80) to detect a difference between lactation groups in mean change in weight for age z-scores of at least 0.28 standard deviation (s.d.) units at 6–9 weeks and 0.31 s.d. units at 6 months. Given an expected standard deviation of z-score of 1.1 [49], the minimum detectable difference in mean z-scores translates to 0.30 and 0.34 at 6–9 weeks and at 6 months, respectively. In Aim 2, for analyses of infant regional adiposity variables at 12 months, the minimal detectable difference in means across lactation categories is 0.29 s.d. units (e.g., 0.20 weight-for-length z-score, 0.86 cm in abdominal circumference, 0.70 mm subscapular, and 2.9 mm sum skinfolds, based on infant data in GDM offspring [29, 30]). In Aim 3, analyses of adiposity in mothers at 6 months and 12 months postpartum, we have sufficient power (0.80) to detect a difference in means of 0.31 s.d. units at 6 months, and 0.29 s.d. units at 12 months (e.g., differences of 2.0 kg/m [2] for BMI, 4.2 cm for waist girth at 6 months, and 1.85 kg/m [2] for BMI, 3.9 cm for waist girth at 12 months). In summary, detectable effect sizes are relatively small, and clinically important.

**Table 2:** Minimum detectable differences in means (standard deviation units, s.d.) for comparison of Intensive (exclusive or mostly) breastfeeding vs. Intensive (exclusive or mixed) formula infant feeding (two-sided test, significance level = 0.05, power = 0.80). [Total number of mother-infant pairs enrolled with one or more study exams; *n* = 466]

Time:	At Birth	6-9 weeks	6 months	12 months
Sample Size	*N* = 466	*N* = 462	*N* = 378	*N* = 423
SD units	0.28	0.28	0.31	0.29

### Data collection and methodology

Table 3 displays a summary of data collection for the SWIFT Offspring study, including variables collected by the parent SWIFT study and the Offspring study. Supplemental infant food intake, physical activity, sleep habits, temperament, infant health and other infant behaviors and characteristics were also assessed via interviews of mothers at study exams.

**Table 3:** SWIFT and SWIFT Offspring studies: description of data elements and timeline

Methodology	Data source	SWIFT Offspring study data collection				Data collection methods
		KPNC Hospital (at birth)	Baseline In-person Exam (6–9 weeks)	Follow-up In-person Exam (6 months)	Follow-up In-person Exam (12 months)	
Infant Anthropometry:	In-person exam	X*	X	X	X	*EMR at birth, and Research quality instruments and standard protocols
Weight (Digital scale, Tanita); Length (Measuring board, Seca);						
Skinfold thicknesses (Holtain calipers) triceps, suprailiac, and subscapular sites; Head and Abdominal circumferences						
Breastfeeding Intensity/duration based on quantitative measures of breast milk and infant formula use;	In-person exam, telephone interviews, mailings	X	X	X	X	Diary birth to 6 weeks, Monthly mailed surveys, Recall past 7 days at in-person exams
Infant Diet History: types and quantity of liquids, milk, sugar-sweetened beverages, juices; monthly introduction of foods (7-day recall, 6-month history)	In-person exam, telephone interviews, mailings	---	X	X	X	7-day recall of diet intake, Dietary history past two age introduced; types and amounts food items
Newborn: birth weight, length, gestational age, sex, Apgar, size hospital stay,	KP EMR VDW	X	---	---	---	Electronic clinical medical records
Maternal Anthropometry: weight, height, waist circumference, % body fat, Bioelectrical Impedance Assessment (BIA) - RWJ	In-person exam	---	X^a^	X	X^a^	Anthropometry using research quality, calibrated instruments and standardized protocols
SES: family income, maternal education, occupation	Surveys	X	---	---	---	SWIFT study
Intrauterine: Pre-pregnancy BMI, GDM severity: 3-h 100 g OGTT, type of GDM treatment, gestational weight gain,	KP Prenatal Care	X	X	---	---	Electronic Clinical databases (EMR) – medical records
Race/ethnicity, delivery method pregnancy complications	KP EMR VDW	X	X	---	---	Surveys, Electronic medical records
Family history of diabetes, paternal BMI, sleep habits, Maternal depression, alcohol, smoking, physical activity, caffeine, dietary intake	Surveys	---	X	---	X	Validated and Standardized questionnaires.
	CES-D					
	PPAQ					
	PrimeScreen					
	Other					
Infant Health – medications, medical conditions	Survey		X	X	X	Standardized questions
Infant Sleep Habits	Survey	--	X	X	X	Sleep habits
Infant Development and Activity	Survey	--	X	X	X	Standardized questions
Temperament (Rothbart scales)	IBQ-R	---	X	X	---	Validated questionnaire
Toddler Behavior Questionnaire (Goldsmith scales)	TBAQ	---	---	---	X	Validated questionnaire

*CES-D* Centers for Epidemiology Studies – Depression Questionnaire, *EMR* Electronic Medical Record, *VDW* Virtual Data Warehouse, *IBQ-R* Infant Behavior Questionnaire – Rothbart, *BIA* Bioelectrical Impedance Assessment, *PPAQ* Postpratum Physical Activity Questionnaire PrimeScreen, *FFQ* Semi-quantitative Food Frequency Questionnaire, *KP* Kaiser Permanente, *TBAQ* Toddler Behavior Assessment Questionnaire, ^a^ = measurements from the parent SWIFT study

* Signifies P <0.05

### The SWIFT study data elements, (women with GDM)

SWIFT participants (mothers delivered of GDM pregnancies) attended three in-person visits (6–9 weeks, 12 months and 24 months) at which time trained research staff obtained anthropometric and body composition measurements, administered surveys to collect behaviors, socio-demographics, and reproductive history, and conducted the 2-h 75 g oral glucose tolerance test (OGTT). Mothers kept diaries and were queried in detail about infant feeding practices (breastfeeding intensity and duration, formula feeding), and infant dietary intake (Table 3). Data collection occurred during telephone interviews in late pregnancy, and at 4–5 weeks and at 6 months post-delivery, as well as monthly mailed surveys from 3–11 months post-delivery and from health plan electronic medical records (EMR) (Fig. 1).

### The SWIFT offspring study (mother-infant pairs), data elements

Study protocol at each exam included anthropometric measurements, and both self-and-interviewer-administered questionnaires. Electronic medical records systems provided key information on perinatal characteristics and pediatric clinical health outcomes. We obtained clinical data on newborn birth weight, length, gestational age, Apgar scores, neonatal intensive care unit (NICU) admissions and health conditions, as well as maternal prenatal 3-h oral glucose tolerance test (OGTT) results, weight at delivery, type of GDM treatment, severity of GDM, and pre-pregnancy weight from health plan EMR. Bilingual English-Spanish research assistants administered the Spanish versions of the consent form and each questionnaire to participants whose preferred language was Spanish. Additionally, validated parent-report measures of infant temperament were collected, as growing evidence supports the putative association between a ‘difficult’ infant temperament and later child obesity [78, 79]. In fact, parents may bottle feed or television to assuage a difficult temperament or negative emotionality [80–83], although this issue has never been studied in the context of GDM offspring.

### Study variables

We present the methodology for measurements of the study variables below including primary outcome measure (i.e., change in infant weight-for-length z-score,WLZ); primary independent variable (i.e., breastfeeding intensity and duration); and intrauterine exposures, sociodemographics and lifestyle covariates. Our selection of covariates was based in part on early life and behavioral risk factors examined in prior studies [6, 74, 84].

### Infant growth – weight-for-length Z-scores

At in-person exams, trained research staff obtained anthropometric measurements (weight, length, health circumference and abdominal circumference) at 6–9 weeks, 6 months and 12 months of age using the WHO Multi-center Growth Reference Study standardized procedures [85]. Weight and length were used to calculate the z-scores to evaluate growth compared to the WHO standard referent population which is based on the growth of healthy breastfed infants and young children raised in environments that do not constrain growth [86]. For children under 24 months of age, use of the 2006 WHO international growth charts is recommended by the Centers for Disease Control and Prevention (CDC) [87, 88]. The primary measure of infant growth is defined as change in infant weight-for-length z-score (WLZ) from birth to 6–9 weeks, 2 to 6 months, and 6 to 12 months of age based on the World Health Organization (WHO) growth charts [87]. We also calculated weight-for-age (WAZ) and length-for-age (LAZ) z-scores to evaluate infant growth at each age.

### Infant feeding assessments

#### Breast milk and formula feeding intensity and duration

Infant feeding practices, including breastfeeding intensity and duration, were assessed prospectively from birth by asking mothers to record amount of formula fed using a weekly diary, and during a telephone interview conducted at 4–5 weeks of age to determine study eligibility. Research staff administered questionnaires to assess infant feeding practices since birth throughout the first year of life during the three in-person study exams, and mothers returned monthly questionnaires by mail. Mothers were asked to report whether they had ever breastfed their child, and if they were currently breastfeeding. If they were not currently breastfeeding, women were asked to specify the date and/or child’s age as well as the reasons for discontinuing breastfeeding. They also reported the frequency of breastfeeding and formula feeding including the number of expressed breast milk feedings by bottle, and formula supplementation (quantity) per 24 h within the past 7 days during each month. Details included the frequency of day and night feedings, frequency of breast milk expression and feeding by bottle, provision of other liquids (water, tea, etc.), use of formula and quantity, type and brand used, use of cereal, sweeteners, other liquids (i.e., Pedialyte), juices, sugar-sweetened water and beverages, and timing of the introduction of solid foods and liquids and the quantities.

At study baseline (6–9 weeks of age), five breastfeeding intensity groups were devised based on cumulative intake since birth: 1) exclusive breastfeeding (0 ounces of formula, and no other liquids); 2) mostly breastfeeding (formula ≤6 ounces of per 24 h; 3) Mixed breast milk and formula (≥7–17 ounces of formula per 24 h) or inconsistent pattern of feeding; 4) mostly formula feeding and some breast milk, (>17 ounces per 24 h); and 5) exclusive formula feeding (no breast milk and less than 3 weeks) [89]. We combined the mixed/inconsistent group with the mostly formula feeding group to configure four infant feeding groups at study baseline.

A combined measure of breastfeeding intensity and duration from birth through one year of age was configured based on the prospectively collected data from the in-person exams, telephone interviews, and monthly questionnaires collected for the parent SWIFT Study. We used the methodology of Piper et al. [90] to calculate a lactation intensity ratio summary score from birth to 12 months. For each month (months 1–12), we calculated a lactation intensity ratio (LIR) based on the number of breastfeedings and the amount and number of milk feedings during an average 24-h period for the previous 7 days (1 week) reported by women. The 24-h recall period is the best method to assess breastfeeding practices because it has greater validity [91] and it minimizes recall error as compared to longer recall periods.

We also collected information on infant dietary intake of supplemental foods/liquids (type, amount, frequency). The LIR was developed for the 1988 National Maternal-Infant Health Survey [90] as an intensity ratio, calculated from the number of breast milk feeds (on average in 24 h) divided by the total number of all milk feeds (on average in 24 h), within a range from 0 to 1.0. Exclusive breastfeeding for a given month received the highest score of 1.0. Exclusive formula feeding received a score of 0 for all time periods. Partial breast-feeding for any month received an LIR of less than 1.0. The scores for lactation were summed across all months until weaning, and an “overall” lactation score was calculated based on the sum of the LIRs for each month over their entire duration of breastfeeding.

The LIR for each month and overall LIR summary score were calculated as follows:

$$ \mathbf{L}\mathbf{I}\mathbf{R}\ \left[\mathbf{n},\ \mathbf{month}\right] = \left(\#\ \mathrm{breastfeedings}\right)\ /\ \left(\#\ \mathrm{breastfeedings} + \#\ \mathrm{formula}\ \mathrm{feedings} + \#\ \mathrm{milk}\ \mathrm{feedings}\right), $$

$$ \mathbf{Overall}\ \mathbf{summary}\ \mathbf{score}\left(\mathrm{sum}\ \mathrm{of}\ 12\ \mathrm{months}\right) = \left[\mathrm{L}\mathrm{I}\mathrm{R}\ 1 + \mathrm{L}\mathrm{I}\mathrm{R}\ 2 + \mathrm{L}\mathrm{I}\mathrm{R}\ 3 + \mathrm{L}\mathrm{I}\mathrm{R}\ 4 + \dots + \mathrm{L}\mathrm{I}\mathrm{R}\ 12\right] $$

### Supplemental dietary intake

Monthly mailed questionnaires asked mothers to report the types of foods and liquids fed to the infant each month from 3 to 11 months of age. At the three in-person exams at 6–9 weeks, 6 months and 12 months of age, research assistants asked mothers to recall the average daily frequency that infants were fed fruit juices, sweetened juices and drinks, sugar added to water or tea, and any cereal in the bottle or other foods fed to the child in the past 7 days. At 6 months of age, a comprehensive dietary history was administered to characterize the dietary intake during the past two weeks, and to assess the transition to supplemental foods and other liquids for each month from birth to 6 months. Mothers reported the types and amounts of foods and liquids consumed, the ages when these were first introduced, and the average dietary history for each month through age 6 months. When the infant reached 12 months of age, mothers also completed a survey about the types of milk, including artificial formula, cow’s milk, soy milk or other milk sources and quantity of milk consumed by the child per 24 h.

#### Anthropometric assessments

##### Infant measurements

Anthropometric measurements for infants were obtained at 6–9 weeks, 6 months and 12 months of age using the procedures developed for the WHO Multicenter Growth Reference Study [86]. Prior to data collection, the research staff were properly trained and certified to follow the WHO standardized procedures for anthropometric measurements [92]. At each in-person exam, trained research assistants measured the infant’s head circumference, abdominal circumference, skinfold thicknesses (at the triceps, suprailiac and subscapular sites), weight and supine length via standardized protocols. Two measurements were obtained during each procedure, with a third measurement obtained if the difference between the first two measurements was greater than 1 cm for length, 0.1 kg for weight, 1 cm for abdominal circumference, and 0.1 mm for skinfold thickness. Measurements of infant weight and length were used to calculate the weight-for-length, weight-for-age, and length-for-age z-scores, and change in z-scores using the WHO Growth Standards (http://www.who.int/childgrowth/en/) as the referent population [92].

Weight was measured on a digital scale (Tanita, Model BD590 infant scale) that calibrates to zero and is accurate to the nearest 5 g. Length was measured to the nearest 0.1 cm using an infantometer (Seca, Model 417 infantometer). Infant head circumference was measured using the Abbot Nutrition of Abbot Laboratories measuring tape. Abdominal circumference was measured to the nearest 1 mm using a tape measure (Gulick Model 67020, ¼ inch measuring tape) made of material that does not stretch and the time of the last feeding episode was recorded. Time of last feeding was recorded for each infant before obtaining the abdominal circumference measurement. Skinfold thicknesses were measured to the nearest 0.2 mm using a Holtain Tanner/Whitehouse Skinfold Calipers which are calibrated at 0 mm at each exam to assess regional adiposity. The sum of the three skinfold measurements was used to evaluate overall body adiposity.

##### Maternal measurements

Maternal body weight and waist circumference were measured at 6–9 weeks, 6 months and 12 months postpartum. Women were weighed on a research quality, calibrated digital scale (Tanita, Model WB110A, 100A) to the nearest 0.1 lb in light clothing and were asked to empty pockets, and remove any heavy jewelry and shoes. Height was measured in bare or stocking feet to the nearest centimeter using a stadiometer (Seca, Model 69072) to the nearest 0.1 in.. Body mass index (BMI) was calculated as weight (kg) divided by height (m) squared and used to evaluate overall adiposity. Waist circumference (waist girth) was measured on a bare abdomen in triplicate to the nearest centimeter at the level of the right ischium using a Gulick *II Plus* 300 cm anthropometric tape (Model 67019). The *Gulick II Plus* tape has a tensioning device attached to the measuring tape that provides a standard amount of tension (4 ounces) while a measurement is being taken.

#### Infant health and behavioral assessments

##### Infant health status

The survey includes questions about the child’s health conditions, current medication use, allergic reactions, hospitalizations since birth, number of teeth the baby currently has, and any serious long-term medical conditions.

##### Infant sleep habits

Infant sleep habits were assessed by a questionnaire that gathered details on where the child sleeps, the position of the child when sleeping, duration of nighttime and daytime sleep (including naps), number of night awakenings per night, longest period of sleep without waking, duration of wakefulness during the night (between 10 p.m. and 6 a.m.), length of time needed to put child to sleep, how the child falls asleep at night (e.g., while feeding, being rocked, etc.), usual sleep and waking time each day, and whether the child’s sleep is considered a problem. The questionnaire was developed in collaboration with Dr. Kathryn Lee [93, 94].

##### Infant development and sedentary activity

A brief questionnaire asked mothers to report the ages when the child could first roll over without assistance, was able to sit up without support, began to crawl and walk without support, and how many teeth developed. Mothers also reported how much time their child spent watching television or videos on a weekly basis.

#### Infant behaviors/temperament questionnaires

##### Rothbart scales

The Rothbart Infant Behavior Questionnaire (IBQ-R) contains 184 items that measures 14 dimensions of infant temperament, including ‘soothability’ and ‘distress to limitations.’ Additionally, a 15-item subscale assesses the child’s gross motor activity, including movement of arms and legs and squirming and locomotor activity. Objective measures of physical activity in infants have not been validated, but qualitative proxy measures of physical activity, such as temperament scales, have been linked with obesity in young children. The IBQ-R was administered at 6–9 weeks and at 6 months of age [95].

##### Goldsmith scale

We utilized the scale to assess child behavior at one year of age. The Toddler Behavior Assessment Questionnaire (TBAQ) contains 108 items that measure six scales of parent-reported temperament-related behavior in 16–36 month old children. A 26-item abbreviated version of the TBAQ was administered at 12 months of age in this study [96]. The scales include activity level, anger, fear, pleasure, and interest. The scale has been validated and internal consistency reliability exceeds .80 for each scale [97].

#### Other risk factors assessments

Using self-and interviewer-administered questionnaires at in-person exams, telephone contacts and monthly mailed brief surveys, we collected data on numerous risk factors, including intrauterine exposures. Women reported family history of diabetes, previous GDM diagnosis, treatment for GDM, and other perinatal complications, newborn outcomes, pre-pregnancy weight, current medical conditions, medication use, pregnancies after enrollment (inter-current pregnancies), and contraception methods, including hormonal contraceptive use.

These questionnaires assessed maternal socio-demographics, medical history, alcohol consumption, smoking, and postpartum depression. Clinical risk factors, including the severity of glucose intolerance during pregnancy utilizing the 3-h 100 g OGTT, were also collected through a variety of methods including the health plan electronic medical records, study phone interviews, in-person surveys and monthly study mailings.

### Other data collection procedures

To streamline the in-person exams, a subset of questionnaires were mailed to mothers at least a week prior to their 6- and 12-month exams and participants submitted the completed questionnaires to research assistants at exams. If the mailed questionnaires were incomplete or not received, they were completed during the in-person exam. For quality control purposes, the questionnaires received by mail were reviewed by research staff for completion and accuracy.

### Quality control procedures

Detailed study operations manuals were developed to standardize the data collection procedures across the study sites. Research staff completed trainings led by the Project Manager which included a series of shadowed study activities for each data collector that were evaluated before the person could begin performing any study activity. The training phase included observation of the staff while conducting their first few measurements with actual study participants. Throughout the study period, refresher trainings involving all data collectors were conducted twice per year.

### Biospecimen collection procedures

Saliva samples were collected from the infants at 6 months of age and older using Oragene DNA kits which were stored at room temperature at the Division of Research for future genetics studies. Saliva samples have been validated as an appropriate method for DNA collection of sufficient quantity and quality for large-scale genetic epidemiological studies [98, 99]. Procedures for the saliva collection from infants involved obtaining written informed consent from the mother. Research assistants checked for consent before performing collection and prepared the Oragene DNA kits. The kits included 2 sponges, a cap, and a collection tube with funnel top. Prior to saliva collection, the mother was asked about the infant’s last feeding time and saliva was collected at least 30 min since last feeding. The research assistants recorded whether a sweetener was used during saliva collection. During collection, infants were kept in an upright position and a sponge was placed in cheek pouch along the gums to soak up as much saliva as possible and gently moved along this area for at least 30 s. The saturated sponge was placed in the V-notch of the funnel and saliva wrung out using a twisting and pushing motion against the inner wall of the V-notch. The procedure was then repeated in the other cheek until the amount collected reached the fill line of the collection tube. Staff received training from the Oragene DNA specialist to ensure proper collection of infant saliva via standardized procedures.

## Discussion and conclusions

Current scientific evidence is mixed regarding the benefits of breastfeeding on future health outcomes for ODM. Breastfeeding has been associated with a lower risk of becoming overweight among ODM in retrospective studies [67, 68], while others found this protective association only among the GDM offspring born to obese mothers [70], or reported a null association [100]. Case–control studies have reported that longer breastfeeding duration may lower the risk of developing type 2 diabetes among ODM offspring [61, 62], but these studies rely on recall of breastfeeding duration. Previous studies of ODM have never conducted prospective assessments of breastfeeding duration, or serial measurements of infant growth, and also have not employed quantitative methods to assess breastfeeding intensity. In one prospective study of ODM, intake of breast milk from diabetic mothers compared with banked breast milk during the neonatal period (first 7 days of life) was associated with adverse health outcomes in children aged 2 years, but was unrelated to type of milk feeding in the subsequent time period [64, 65].

Finally, limitations of all studies, except one, include the recall of any or exclusive breastfeeding duration several years to decades later by mothers of older children or adult offspring and relatively small sample sizes that provide inadequate statistical power. Moreover, the measures of infant feeding did not use quantitative methods to assess breastfeeding intensity, and almost all studies lack sufficient control for potential confounders including parental characteristics, prenatal and postnatal exposures and perinatal outcomes.

The SWIFT Offspring study is the first to conduct prospective and quantitative assessments of breastfeeding intensity and duration, and infant supplemental food intake and behaviors, as well as longitudinal growth measurements and changes in adiposity during the first year of life among the offspring born to a large, well-characterized cohort of women with a GDM pregnancy. The study also assessed changes in overall adiposity among women with recent GDM. This observational study accounts for intrauterine metabolic exposures, postnatal behaviors, and genetic influences represented by parental body size, as well as sociodemographic risk factors. The careful control of potential confounders is intended to minimize bias from reverse causation or unmeasured confounding. The design is a robust alternative to the randomization of mother-infant pairs infant feeding groups which is not feasible or desirable for this high-risk group. Identification of modifiable risk factors that influence postnatal programming of adiposity, appetite, and/or energy regulation mechanisms among GDM offspring is necessary to formulate strategies for prevention of obesity and type 2 diabetes mellitus in this high-risk group. The SWIFT Offspring study will significantly advance our current knowledge about the effects of postnatal feeding on growth during infancy among GDM offspring. This study also lays the foundation for future studies to evaluate the impact of breastfeeding on the GDM offspring’s long-term risk of obesity and diabetes.

## Abbreviations

BMI: Body Mass Index
CDC: Center of Disease Control and Prevention
CI (statistics): Confidence Interval
EMR: Electronic Medical Records
FFQ: Food Frequency Questionnaire
GDM: Gestational Diabetes Mellitus
IBQ-R: Rothbart Infant Behavior Questionnaire
KPNC: Kaiser Permanente Northern California
NICHD: National Institute of Child Health and Human Development
ODM: Offspring of Diabetic Mothers
OGTT: Oral Glucose Tolerance Test
OR: Odds Ratios
PAQ: Physical Activity Questionnaire
SWIFT: Study of Women, Infant Feeding, and Type 2 Diabetes After GDM Pregnancy
SWIFT Offspring: Study of Women, Infant Feeding and Type 2 Diabetes After GDM Pregnancy and Infant Growth in their Offspring
TBAQ: Toddler Behavior Assessment Questionnaire
VDW: Virtual Data Warehouse
WHO: World Health Organization
WLZ: Weight-for-Length Z-score
